# The year that shaped the outcome of the OspA vaccine for human Lyme disease

**DOI:** 10.1038/s41541-022-00429-5

**Published:** 2022-01-27

**Authors:** Raymond J. Dattwyler, Maria Gomes-Solecki

**Affiliations:** 1 Department of Microbiology and Immunology, New York Medical College, Valhalla, NY USA; 2 Department of Microbiology, Immunology, and Biochemistry, University of Tennessee Health Science Center, Memphis, TN USA

**Keywords:** Immunology, Vaccines

## Abstract

The expansion of Lyme borreliosis endemic areas and the corresponding increase of disease incidence have opened the possibility for greater acceptance of a vaccine. In this perspective article, we discuss the discovery of outer surface protein A (OspA) of *B. burgdorferi*, and the subsequent pre-clinical testing and clinical trials of a recombinant OspA vaccine for human Lyme disease. We also discuss in detail the open public hearings of the FDA Lyme disease vaccine advisory panel held in 1998 where concerns of molecular mimicry induced autoimmunity to native OspA were raised, the limitations of those studies, and the current modifications of recombinant OspA to develop a multivalent subunit vaccine for Lyme disease.

## Lyme disease and outer surface protein A (OspA) of *B. burgdorferi*

The skin lesion erythema migrans is the classic clinical marker of early Lyme disease. The first well documented case of erythema migrans acquired in the United States was reported in 1970 in Wisconsin^1^. In 1976, Mast and Burrows described a cluster of cases of erythema migrans in Southeastern Connecticut^2^. A year later, others described a cluster of patients with large joint arthritis in the area of Old Lyme, Connecticut^3^, and named this condition Lyme arthritis. After retrospective analysis, it became apparent that most of these patients previously had erythema migrans and some had heart block, facial nerve palsy, or meningitis. At that point, the name of the illness was changed from Lyme arthritis to Lyme disease^4^.

*Borrelia burgdorferi* was identified as the etiologic agent of this illness after a spirochete was isolated from *Ixodes dammini* ticks^5^ (currently named *I. scapularis*) and from blood of patients with Lyme disease^6^. The earliest reference to an outer surface protein of *Borrelia burgdorferi* that associated with bacterial agglutination dates to 1984^7^. In vitro culture^8^ and immunochemical analysis of this spirochete soon followed^7^, as did subsequent recombinant cloning of the *ospA* gene^9^. OspA was proposed as a vaccine candidate for Lyme borreliosis after anti-OspA antibodies^10,11^ and immunization with recombinant OspA protein (rOspA)^12^ protected mice from challenge with several strains of cultured *B. burgdorferi*. Additional studies showed that *B. burgdorferi* was eliminated from infected nymphal ticks feeding on rOspA vaccinated mice and monkeys^13,14^. Following rOspA vaccination, blockage of transmission of the spirochete from the tick vector to the host^15^ and the ability of anti-rOspA antibody to agglutinate *B. burgdorferi*^7,16^ suggested a bactericidal mediated mechanism of action. However, the titer of anti-rOspA antibody required to eliminate *B. burgdorferi* from Ixodes ticks was 2 Logs higher than the titer required to block transmission^17^. Although *B. burgdorferi* attachment within feeding ticks was dependent on OspA^18^ and a receptor for OspA (TROSPA) was found in the *I. scapularis* midgut, blocking TROSPA resulted in a diminished but persistent *B. burgdorferi* colonization of the tick midgut^19^. Further studies using OspA-specific monoclonal antibodies showed that even low concentrations of antibody blocked transmission despite the presence of many live spirochetes in the tick^20^. Thus, the mechanism of action mediated by bactericidal-independent OspA antibody that results in blockage of transmission of *B. burgdorferi* still needs to be clarified.

The clinical picture of Lyme disease has changed over the past 30 years. Lyme arthritis was emphasized as the key manifestation of late disease in North America and it may have been more common in the 1980s, but it is not common now. With early diagnosis and more effective treatment protocols the incidence of true Lyme arthritis dropped dramatically^21–23^. Although arthritic manifestations are still frequently reported, subjective joint pain (arthralgias) and true arthritis are conflated, which leads to an overestimation of Lyme arthritis incidence. In a recent Canadian study of 1230 patients reported to have Lyme disease, the overall incidence of arthritis was 0.028%. Of the 475 cases reported to have Late Lyme disease only 35 (7.4%) manifested true arthritis, while 440 (92.6%) had arthralgias^24^. If we consider the estimate that about 10% of patients who develop Lyme arthritis will develop treatment-resistant Lyme arthritis (i.e., antibiotic-refractory Lyme arthritis)^25^ an objective estimate of current (2021) treatment-resistant Lyme arthritis incidence in Lyme disease patients would be about 0.0028%.

In the mid-1990s, when recombinant OspA (rOspA) was being tested in clinical trials as a potential vaccine candidate for human use it was unknown if this protein was involved in the pathogenesis of treatment-resistant Lyme arthritis. In a retrospective study of the antibody response to infection with *B. burgdorferi*, significant levels of anti-OspA/OspB antibodies were not present in patients with erythema migrans or meningitis; in contrast, in patients with prolonged disease, some of which were previously treated with antibiotics and later developed Lyme arthritis, 71% had measurable antibodies to native OspA and OspB^26^. Another study reported that OspA reactive Th1 cells were detectable in synovial fluid of treatment-resistant Lyme arthritis patients years after antibiotic treatment but were not detectable in the joints of patients with treatment-responsive Lyme arthritis^27^. Although spirochetal DNA was found in the joint before most Lyme arthritis patients underwent antibiotic treatment, no spirochetal DNA was found in the joints of patients after antibiotic treatment^28^. This implied that in the treatment-resistant Lyme arthritis patient population, joint inflammation persists even after the apparent eradication of the spirochete from the joint with antibiotics. Individuals with treatment-resistant Lyme arthritis were more likely to have the major histocompatibility complex class II (MHC II) cell surface receptor encoded by the human leukocyte antigen allele (HLA) DR4B1*0401 or DR4B1*0101^26^. The DRB1*0401-predicted dominant epitope was identified as a short epitope in *B. burgdorferi* OspA (aa165–183)^29^ that is close to a T cell helper epitope formerly identified in the carboxyl terminus of the protein^30^. The amino acid sequence of this peptide was partially homologous to a sequence of the human leukocyte function-associated antigen-1 (hLFA-1α_L322–340_) (Table 1) that was also shown to bind strongly to DRB1*0401^29^. Given that synovial fluid T cells of 6 out of 11 treatment-resistant Lyme arthritis patients produced IFNγ when stimulated with OspA_165–183-_peptide and that 5 of those 6, also produced IFNγ when stimulated with hLFA-1α _L332–340_-peptide, it appeared plausible that a cross-reactive autoimmune event might drive the inflammatory response in the joint in some HLA-DRB1*0401 individuals^29^. This molecular mimicry hypothesis predicted that after entry of *B. burgdorferi* into the joint, T cells that recognize a microbial antigen peptide (in this case, OspA_165–183_) and T cells that recognize a related self-peptide (in this case, LFA-1α _L332–340_) produce IFNγ that upregulate expression of ICAM-1 on synoviocytes as well as MHC class II molecules on local antigen-presenting cells, and these events lead to the recruitment of more LFA-1 expressing cells. Thus, the subsequent enhanced presentation of self-peptides augments and propagates the local inflammatory response even after *B. burgdorferi* has been cleared^31^. An under-appreciated limitation of the molecular mimicry hypothesis is the concept of T-cell recognition degeneracy. The structure and functioning of the T cell receptor (TCR) were characterized during the late 1990’s. It was known that T cell receptors recognize the complex of peptides bound to HLA. In 1998, a number of impactful papers showed that the TCR must be promiscuous to accommodate the enormous repertoire of antigens they are presented with. In addition to a high level of non-specificity essential to the proper functioning of T cell receptors^32^, it was shown that binding is primarily dependent on the shape created by conformation changes when the MHC captures the peptide in the binding groove and that amino acid sequence homology is a poor predictor of binding^33,34^. In 2000, Maier and al. examined the recognition of self-antigens by HLA-DR4-restricted T cells specific for peptides of *B. burgdorferi* OspA and found extensive cross-reactivity between T cells reactive to the OspA_165–173_ epitope and many supertope-matching peptides from human proteins. They concluded that T-cell cross-reactivity is a common phenomenon and that T cell cross-reactive epitopes alone do not predict molecular mimicry-induced autoimmune disease^35^. Further weakening the proposed molecular mimicry hypothesis is that both DRB1*0401 and DRB1*0101 alleles are frequently associated with many other diseases, namely rheumatoid arthritis^36^, that do not have an infectious etiology. Another limitation of the molecular mimicry hypothesis is that co-infections or other inflammatory syndromes may lead to increased production of IFNγ by Th1 cells in the joint that will upregulate enhanced presentation of self-peptides that amplify local inflammatory responses. Whether differences in OspA natively expressed in *B. burgdorferi* and recombinant OspA produced in expression systems could account for differences in inflammation needs further investigation.

**Table 1:** Sequence homology between human fleucocyte function associated Antigen-1 (hLFA-1) and *B. burgdorferi* native OspA.

Protein	Amino acid sequence
hLFA-1α_L332–340_	**Y V** I **E G T** S K Q
BbB31 OspA_165–173_	**Y V** L **E G T** L T A

hLFA-1a_L332–340_, human leukocyte function associated antigen-1, amino acid residues 332–340; BbB31 OspA_165–173_, *Borrelia burgdorferi* sensu stricto (B31) outer surface protein A, amino acid residues 165–173; **bold**, conserved residues.

## Clinical trials of the recombinant OspA (rOspA) vaccine for Lyme disease prevention

The results of a Phase II clinical trial were published in 1994. It reported on the safety and immunogenicity of recombinant OspA (rOspA) with and without adjuvant in 36 healthy adult volunteers. The researchers found that both vaccine compositions induced high-titer of anti-rOspA antibodies that neutralized *B. burgdorferi* in vitro, with the most common adverse reactions being pain and tenderness at the site of inoculation^37^. The safety and immunogenicity of the rOspA vaccine was again tested in 30 healthy volunteers who had been previously diagnosed with Lyme disease. In that study, reported in 1995, 93% of subjects developed high titer of antibody to rOspA and transient systemic side effects were recorded with three subjects also reporting mild arthralgias that lasted 24 h^38^. Between January of 1995 and March of 1998 two Phase III efficacy studies were done in which two slightly different compositions of recombinant OspA were tested. In one study, a chemically lipidated full length recombinant OspA protein was adsorbed to aluminum hydroxide (L-rOspA with adjuvant, LYMErix^TM^, SmithKline Beecham (SKB), Pittsburgh, PA, now GlaxoSmithKline—GSK) and tested in a placebo-controlled trial: 5469 subjects received the vaccine and 5467 subjects received a non-OspA placebo^39^. In the other study, also a placebo-controlled trial, full length recombinant OspA lipoprotein was tested without adsorption to any adjuvant (ImuLyme^TM^, PasteurMérieux-Connaught, Swiftwater, PA). In that study, 5156 subjects received the vaccine and 5149 subjects received a non-OspA placebo^40^. In the first study, two IM inoculations of adjuvanted L-rOspA vaccine (LYMErix^TM^) prevented Lyme disease with 49% efficacy in the first year, and a third IM inoculation a year later prevented infections with 76% efficacy^39^. In the second study, two IM inoculations of non-adjuvanted rOspA lipoprotein (ImuLyme^TM^) prevented Lyme disease with 68% efficacy in the first year, and a third IM inoculation prevented infections with 92% efficacy in the second year^40^. It is possible that differences in efficacy of both vaccines could be due to differences in the composition. The immune response to OspA has been shown to be dependent on lipid modification of this protein^41^. The lower efficacy rate of LYMErix^TM^ compared to ImuLyme^TM^ may have been related to the chemical lipidation process of the purified protein. In contrast, the ImuLyme^TM^ composition was purified as a lipoprotein and was used without adsorption to adjuvant.

In both LYMErix^TM^ and ImuLyme^TM^ clinical trials, a thorough analysis of adverse effects was performed. In both, administration of the vaccine was associated with mild to moderate local and systemic reactions that lasted 3–7 days and there was no significant increase in the frequency of arthralgias, arthritis, or neurologic events in vaccine recipients in comparison to placebo controls^39,40^. Although causality was not shown, a later case report highlighted that transient symmetrical polyarthritis was observed in two males over 40 years of age as a possible adverse event of rOspA vaccination. In both cases, the adverse event was successfully treated with a 5-day course of ibuprofen^42^. In the meantime, researchers found that rOspA vaccine efficacy was dependent on the maintenance of high antibody titers in serum over time^43,44^. Furthermore, others found that vaccine-induced immune responses to rOspA did not replicate the sequence of events needed in natural infection to induce treatment-resistant Lyme arthritis^45^. More recent systematic reviews and meta-analysis of published data have found that reported adverse events were not different between vaccinated and placebo groups^46,47^.

## The first meeting of the FDA Lyme disease vaccine advisory panel: May 1998

SmithKline Beecham (SKB) decided to move forward with the LYMErix vaccine for the prevention of Lyme disease and submitted an application to the FDA. In May of 1998, the FDA officers reviewing the application along with a panel of selected FDA advisers, the Vaccines and Related Biological Products Advisory Committee, participated in a public, FDA-sponsored meeting to discuss the LYMErix vaccine. A transcript of the meeting is available^48^. A pre-meeting package included details on the vaccine clinical study carried out by SKB. The company’s representatives gave an overview of the study including the study design, efficacy, and safety data. During the safety discussion, the study’s lead investigator presented previously undisclosed data suggesting that *B. burgdorferi* entry into the joint could induce autoimmune arthritis in genetically susceptible individuals due to molecular mimicry between a dominant T cell epitope in *B. burgdorferi* native outer surface protein A and the human leukocyte function-associated antigen 1 within the pro-inflammatory milieu of the joint^48^. Because the members of the advisory panel had not been briefed on these new scientific developments, they did not have an opportunity to gather evidence beforehand to help the panel understand the issue. Although it was reinforced that natural infection, not vaccination with rOspA, may play a role in treatment-resistant Lyme arthritis in a very small percentage of genetically predisposed individuals, the suggestion that there could be cross-reactivity between a human integrin and OspA raised concerns, there were discussions of unanticipated potential risks of LYMErix, as well as a need for greater caution and continued testing. The consensus of the panel was that the study data did not show significantly different safety issues among subjects in the vaccine and in the placebo groups, and that a Lyme disease vaccine would benefit public health. Thus, the panel recommended to the FDA that LYMErix should be approved. Other factors that diminished the enthusiasm for the vaccine were the low efficacy rate of LYMErix in the first year, the need for continued booster doses to maintain sufficient titer of neutralizing antibodies, the availability of effective treatment and that children were not included^48^. In January of 2001, another FDA meeting was held. A perspective on that meeting is discussed elsewhere^49^. The FDA never withdrew the SKB license to commercialize the OspA vaccine.

Editorial reviews on the demise of the Lyme disease vaccine have been written^49–55^ two of which^54,55^ discuss risk communication and policy implications. We agree that scientific evidence and best patient care practices should guide the ethics of Lyme disease activism. However, we also acknowledge that unclear, sometimes contradictory scientific terminology may have led to confusion that drove health care professionals’ vaccine hesitancy and subsequent public skepticism.

## Current recombinant OspA (rOspA) based vaccines

After the 1998 Vaccines and Related Biological Products Advisory Committee FDA meeting, researchers started working on strategies to re-engineer rOspA as a vaccine candidate. The objective was to modify the epitope in rOspA identified as a putative mimic of hLFA1 while preserving the integrity and immunogenicity of the protein. Some modified the epitope by site directed mutagenesis, while others swapped the putative sequence with the same region of another genospecies such as *B. afzellii*. In both cases, the mutated rOspA protected mice from needle^56^ and tick transmitted^57^ *B. burgdorferi* infection. rOspA chimeras containing different sequences of Borrelia genospecies were constructed with the ultimate goal of developing a vaccine applicable to both the US and the European market^58^. The intellectual property covering sequence substitutions in the C terminus of full length rOspA with the equivalent sequences from *B. garinii* and *B. afzelli* was eventually licensed by Stony Brook University to Baxter^58^. This license then originated the multivalent six serotype rOspA compositions further developed by Baxter scientists. Results of the Baxter Phase I and Phase II clinical trials published in 2013 showed that the updated formulation was both safe and immunogenic^59^. Subsequently, Valneva Austria GmbH applied a similar strategy to produce a composition containing only the modified C-terminus domains^60^ of 6 serotypes of rOspA to develop a subunit multivalent broadly protective vaccine (VLA15)^61^. VLA15 is currently undergoing two Phase II clinical trials to determine the best dose (573 subjects) and schedule of immunization (246 subjects) for human use^62^. VLA15 technology was acquired by Pfizer in April of 2020^63^ and both companies are collaborating to codevelop and commercialize their Lyme disease vaccine. Other strategies to use rOspA based prevention measures have been described elsewhere^64–66^.

## Conclusions

Two recombinant OspA vaccines have been proven efficacious for human use and new candidates are in development. The initial hypothesis that native *B. burgdorferi* OspA may contribute to the development of treatment-resistant Lyme arthritis was scientifically questionable, but it raised safety concerns regarding recombinant OspA vaccines. Nearly two decades after the Lyme disease vaccine was withdrawn from the market, there continues to be a lack of evidence that recombinant OspA induces clinically significant cross-reactivity with the human hLFA-1 epitope.

## Data Availability

Data sharing not applicable to this article as no datasets were generated or analyzed during the current study.
